# Life Years Lost Associated with Obesity-Related Diseases for U.S. Non-Smoking Adults

**DOI:** 10.1371/journal.pone.0066550

**Published:** 2013-06-18

**Authors:** Su-Hsin Chang, Lisa M. Pollack, Graham A. Colditz

**Affiliations:** 1 Division of Public Health Sciences, Department of Surgery, Washington University School of Medicine, St. Louis, Missouri, United States of America; 2 George Warren Brown School of Social Work, Washington University in St. Louis, St. Louis, Missouri, United States of America; National University of Singapore, Singapore

## Abstract

The objectives of this paper are to predict life years lost associated with obesity-related diseases (ORDs) for U.S. non-smoking adults, and to examine the relationship between those ORDs and mortality. Data from the National Health Interview Survey, 1997–2000, were used. We employed mixed proportional hazard models to estimate the association between those ORDs and mortality and used simulations to project life years lost associated with the ORDs. We found that obesity-attributable comorbidities are associated with large decreases in life years and increases in mortality rates. The life years lost associated with ORDs is more marked for younger adults than older adults, for blacks than whites, for males than females, and for the more obese than the less obese. Using U.S. non-smoking adults aged 40 to 49 years as an example to illustrate percentage of the life years lost associated with ORDs, we found that the mean life years lost associated with ORDs for U.S. non-smoking black males aged 40 to 49 years with a body mass index above 40 kg/m^2^ was 5.43 years, which translates to a 7.5% reduction in total life years. White males of the same age range and same degree of obesity lost 5.23 life years on average – a 6.8% reduction in total life years, followed by black females (5.04 years, a 6.5% reduction in life years), and white females (4.7 years, a 5.8% reduction in life years). Overall, ORDs increased chances of dying and lessened life years by 0.2 to 11.7 years depending on gender, race, BMI classification, and age.

## Introduction

The increasing prevalence of overweight and obesity has caused an increased risk of obesity-related comorbidities. These obesity-related diseases (ORDs) include serious chronic diseases, such as coronary heart disease, hypertension, type 2 diabetes, stroke, dyslipidemia, and some cancers, such as endometrial, breast, colon cancers [1], [2], and multiple myeloma [3]. The rising trend of ORDs has not only increased annual medical spending [4], [5] but also increased the risks of mortality. The Centers for Disease Control and Prevention (CDC) estimates that approximately 112,000 deaths are associated with obesity each year in the United States [6], [7].

A large body of literature has studied the relationship between mortality and obesity [8]–[27]. However, most of the studies did not take ORDs into consideration, even though obesity-attributable comorbidities have been known to add variation to the simple relationship between mortality risk and body mass index (BMI – the ratio of weight in kilograms to the height in meters squared) [25], [26], [28]–[32]. In terms of life years measurement, Stevens et al. [27], Peeters et al. [11], Fontaine et al. [14], and, recently, Finkelstein et al. [33] attempted to calculate life years lost to obesity, but did not estimate the change in the life years associated with ORDs. It is known that not all obese individuals are at a higher health risk. Moreover, obesity per se does not cause death directly; it is those diseases associated with obesity that shorten life years. To our knowledge, none has focused on the incidence of ORDs or the association of ORDs with mortality and life expectancy.

Furthermore, most of the published literature studying the association between BMI and mortality employed Cox Proportional Hazards (PH) models [34]. The problem with using the Cox PH models is that they do not take into account unobserved individual heterogeneity, e.g., familial risks, which affects the outcome; however those data were unavailable to investigators. Evidence has shown that failing to control for unobserved heterogeneity results in biased estimation and, consequently, biased hazard ratios and leads to erroneous conclusions [35]–[39]. Our study used the Mixed Proportional Hazard (MPH) model [37], which takes unobserved heterogeneity into account and therefore presumably provides a more precise estimation [35]–[38] than the Cox PH models. The MPH model was preferred in the duration analysis because individuals with relatively high hazard rates for unobserved reasons (e.g., people who have family history of cancer(s) and genetic abnormalities) die earlier on average. Consequently, samples of survivors are selected [36]. Inferences based on these selected samples are likely to be biased. We further extended the estimation results to simulate life years for the group of individuals with the same characteristics and calculate the life years lost associated with ORDs. The simulation approach we employed provides an alternative to the life table approach to project life years.

The aims of this study are (i) to examine the relationship between ORDs and mortality by using the MPH models and data from a national probability sample of the U.S. civilian noninstitutionalized population; and (ii) to predict life years lost associated with ORDs using simulated cohorts of the population. The projection of life years lost is based on the observed characteristics of the sample between 1997 and 2000 as a snapshot of their life span, and their characteristics upon survey determines their predicted life years. Our purpose is to investigate how ORDs in general observed at different life stages will impact life years for the target populations, rather than to examine ORDs separately.

## Materials and Methods

### The Data

Our data were extracted from (i) the National Health Interview Survey (NHIS) [40]; and (ii) the NHIS Linked Mortality Public-use Files [41]. The NHIS is a multi-purpose health survey providing health information on the civilian, noninstitutionalized, household population of the United States. The NHIS consists of three major components: Family, Sample Adult, and Sample Child. From each family in the survey, one sample adult and one sample child (if any children under age 18 are present) are randomly selected, and information on each is collected from the sample adult core and the sample child core questionnaires [42]. This study used the Sample Adult Data Files, which contain data on adults aged 18 years and older.

Mortality data were extracted from the NHIS Linked Mortality Public-use Files [41], which provide mortality follow-up data for the NHIS sample from the date of interview through December 31, 2006 [43]. We linked the individual data in the NHIS Sample Adult Data Files to the data in the NHIS Linked Mortality files by personal identification numbers.

The sample in our study was retrieved from NHIS years 1997 to 2000 [44], and the exclusion criteria were as follows: (i) individuals with any missing data on the target variables; (ii) individuals smoking over 100 cigarettes in their entire life, because analyses can be confounded by illnesses associated with smoking [20], [21], [45]; (iii) women pregnant at the time of survey, because BMI levels are unstable during pregnancy; and (iv) patients who have ever been diagnosed with cancer or a malignancy of any kind, because their BMI levels are less stable due to the cancer treatments and appetite loss.

### Outcome Variable, Covariates, and Model Specifications

Age at death or censor was the outcome variable. We included the following covariates in each model: gender, race, educational attainment, alcohol consumption, and physical activities. For race, we used dichotomized variables for whites and blacks. We controlled for educational attainment by using a binary variable to indicate whether the individual is a high school graduate. Alcohol consumption is dichotomized by whether an individual had had no more than 12 drinks of any type of alcoholic beverage in the individual’s entire life upon survey. Physical activity was dichotomized by whether the individual was engaged in modest or vigorous physical activities for 10 minutes at least once per week.

We also examined variables containing ORD information and constructed an ordinal variable recording the number of ORDs. The ORDs in our study included coronary heart disease, hypertension, diabetes, and stroke [46]. In addition, we generated age dummies delineating an individual’s age at survey. We also considered a binary variable recording whether individuals reported that they had at least one ORD. Age dummies included individuals between ages 20 and 29, 30 and 39, 40 and 49, 50 and 59, 60 and 69, and 70 and above at survey. The reference group was individuals below 20 years of age.

We considered BMI classifications based on the standards established by the World Health Organization [47]: underweight for people whose BMI is less than 18.5 kg/m^2^; normal if BMI ∈ [18.5, 25); overweight if BMI ∈ [25, 30); class I obese if BMI ∈ [30, 35), class II obese if BMI ∈ [35, 40), and class III obese if BMI is at least 40 kg/m^2^. We also considered BMI level of linear, quadratic [14], [48], and inverted forms [14], [19], [22].

We tested different model specifications and parametric assumptions. For the parametric specification, we assumed different distributions of unobserved heterogeneity and changed the number of parameters for the baseline hazard. Different combinations of model specifications and parametric assumptions were performed. All model specifications considered in our study are listed in Table S1 in Appendix S1. Our final model was determined by a weighted Akaike information criterion (WAIC) because of the complex sampling design in the NHIS data [49]. The following covariates were included in the final model: male, white, black, high school graduate, the number of ORDs, alcohol consumption, physical activity, BMI classifications in the form of binary variables (underweight, overweight, class I, II, and III obese, where normal-weight was considered the base group), age dummies, and the interaction terms of age dummies with the number of ORDs. Model estimation is detailed in Appendix S1.

### Prediction of Life Years Lost Associated with ORDs

Life years lost was predicted by simulating life years for populations with at least one ORD and without ORDs based on the estimates in the final model. We divided our sample into groups with different combinations of race, gender, age, and BMI classification. For each group, we simulated their survival densities based on the parameter estimates in the final model and predicted life years. Life years lost was projected by comparing the predicted life years of people with ORDs to that of people without ORDs within the same group. The bootstrap method [50], [51] was performed to resample the subpopulations 1,000 times in order to compute the means and standard errors.

All estimations and bootstraps were adjusted for the complex sampling design [50]–[53] in the NHIS [42]. STATA (11, Stata Corp, College Station, TX) was used to obtain the summary statistics for the sample and population, and MATLAB (7.13, R2011b, MathWorks Inc, Natick, MA) was used to perform estimations and simulations.

### Sensitivity Analyses

A probabilistic sensitivity analysis was conducted to explore the variation in life years lost prediction arising from the parameter uncertainty in the simulations [54]. We first sampled the parameters from the distribution of our estimators [52]. For each set of parameters, we simulated life years and computed the life years lost associated with ORDs for each subgroup. We repeated this process 1,000 times and computed the means and standard errors [55].

### Hazard Ratios for Death

We computed the changes in the risk of death at different life stages associated with an additional ORD. These marginal effects of ORDs on hazard rate enabled us to explore how an additional ORD impacted mortality.

We also computed hazard ratios for BMI classifications by dividing the death rates for the underweight, overweight, class I, II, and III obese by the death rates for the normal-weight. These hazard ratios allowed us to compare and contrast our findings with those in previous studies that used other approaches and models.

## Results

### Descriptive Statistics

Table 1 presents the summary statistics of our sample and the estimated population. The sample contained 61,873 individuals, representing a population of 93,853,798 U.S. non-smoking adults. Among the sample, 38% were male, 75% were white, and 16% were black. Almost 80% of the sample had a high school degree. 4,017 deaths were identified. The mean age at death was 77 years. The maximum age in our sample was 94 years. The largest percentage of people (28%) died between 85 and 89 years of age; 476 people died at age 90 or older.

**Table 1:** Descriptive statistics for U.S. non-smoking adults in the sample and the population, 1997–2000.

Variable	n^a^ (%)				N^b^ (%)				Variable		n^a^ (%)				N^b^ (%)			
**Total**	61,873				93,853,798				**Phy. Act** ^f^		31,217			(50.45)	51,464,802		(54.84)	
**Gender**									**Age at survey**									
Male	23,518			(38.01)	41,383,588		(44.09)		19−		2,174			(3.51)	5,095,174		(5.43)	
Female	38,355			(61.99)	52,470,210		(55.91)		20–29		12,865			(20.79)	20,715,096		(22.07)	
**Race**									30–39		15,047			(24.32)	22,390,794		(23.86)	
White	46,598			(75.31)	73,640,855		(78.46)		40–49		11,742			(18.98)	18,765,606		(20.00)	
Black	9,679			(15.64)	12,379,346		(13.19)		50–59		7,078			(11.44)	10,746,130		(11.45)	
**Education**									60–69		5,152			(8.33)	7,028,974		(7.49)	
High school^c^	49,082			(79.33)	78,561,270		(83.71)		70+		7,815			(12.63)	9,112,022		(9.71)	
Under	12,791			(20.67)	15,292,528		(16.29)		**Age at death**									
**BMI Classification**									29−		46			(1.15)	89,986		(1.90)	
Underweight	1,304			(2.11)	2,023,392		(2.16)		30–34		36			(0.90)	54,090		(1.14)	
Normal weight	27,345			(44.20)	40,070,478		(42.69)		35–39		50			(1.24)	73,094		(1.55)	
Overweight	20,492			(33.12)	32,360,352		(34.48)		40–44		85			(2.12)	119,106		(2.52)	
Class I obese	8,406			(13.09)	12,867,565		(13.71)		45–49		120			(2.99)	178,146		(3.77)	
Class II obese	2,773			(4.48)	4,246,025		(4.52)		50–54		118			(2.94)	154,952		(3.28)	
Class III obese	1,553			(2.51)	2,285,986		(2.44)		55–59		112			(2.79)	154,217		(3.26)	
**ORDs** ^d^	15,178			(24.53)	20,772,263		(22.13)		60–64		156			(3.88)	194,259		(4.11)	
0	46,695			(75.47)	73,081,535		(77.87)		65–69		209			(5.20)	276,619		(5.85)	
1	12,003			(19.40)	16,680,111		(17.77)		70–74		299			(7.44)	381,127		(8.06)	
2	2,642			(4.27)	3,434,897		(3.66)		75–79		468			(11.65)	556,254		(11.76)	
3	474			(0.77)	578,575		(0.62)		80–84		698			(17.38)	766,960		(16.22)	
4	59			(0.10)	78,679		(0.08)		85–89		1,144			(28.48)	1,231,868		(26.05)	
**No alcohol** ^e^	7,173			(11.59)	10,464,992		(11.15)		90+		476			(11.85)	498,720		(10.55)	
			**n**			**N**		**Minimum**		**Maximum**			**Mean**			**Standard deviation**		
**BMI (kg/m^2^)**			61,873			93,853,798		6.6		89.9			29.77			16.10		
**Age at death (years)**			4,017			4,729,398		20.0		94.0			76.57			15.01		

Source: Authors’ calculations based on data from National Health Interview Survey, 1997–2000.

Notes: a. n: sample size; b. estimated population size; c. high school graduates; d. ORD: obesity-related diseases; e. the individual had had no more than 12 drinks of any type of alcoholic beverage in the individual’s entire life; f. the individual engaged in physical activities for 10 minutes at least once per week.

The average BMI of the sample was 29.77 kg/m^2^; 2% of the sample were underweight; 44% of the sample had a BMI within the normal-weight range; 33% were overweight; 13%, 5%, and 3% of the sample belonged to class I, II, and III obese, respectively. Approximately 75% of the sample reported no ORDs before the survey; 19% of the sample reported that they had one ORD; 4% had two ORDs; and less than 1% of the sample had at least three ORDs. About 12% of the sample reported that they had no more than 12 drinks of any type of alcoholic beverage in their entire life, and more than half of the sample engaged in vigorous or moderate physical activity for 10 minutes at least once per week.

### Life Years Lost Associated with ORDs

**Figure 1:**
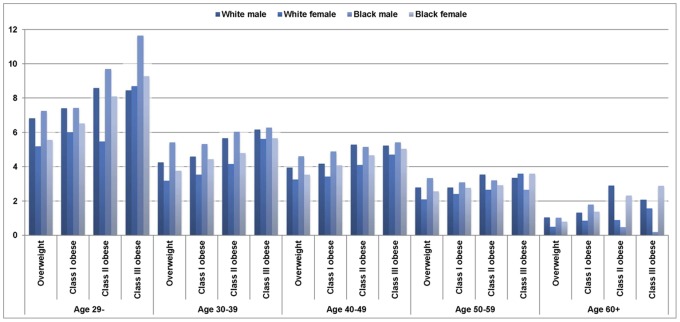
Life years lost associated with obesity-related diseases for U.S. white and black, male and female, overweight and obese, non-smoking adults.

Figure 1 presents the patterns of life years lost associated with ORDs for U.S. white and black, male and female, non-smoking adults who were at least overweight. Table 2 shows life years lost associated with ORDs for all race-gender-age-BMI classification cohorts of U.S. non-smoking adults. In general, the younger an adult developed ORDs, the more life years were lost associated with the comorbidities. For blacks and whites who were overweight or obese, ORDs were expected to decrease life years from 5.20 (overweight white female) to 11.65 (class III obese black male) for people under 29 years of age, while ORDs were expected to decrease life years from 0.20 (class III obese black male) to 2.92 (class II obese white male) for people over 60 years of age (Table 2), depending on degree of obesity, gender, and race.

**Table 2:** Predicted life years lost associated with obesity-related diseases for U.S. non-smoking adults, 1997–2000^a^.

		White male		White female		Black male		Black female		Other male		Other female		Overall	
**29**−	**Underweight**	6.88	(8.71)	6.75	(4.70)	–	(–)	7.74	(6.67)	10.12	(11.67)	6.54	(5.52)	7.11	(3.03)
	**Normal-weight**	6.54	(1.57)	5.92	(1.46)	7.48	(3.95)	6.57	(2.12)	6.53	(4.08)	5.06	(2.87)	6.13	(0.89)
	**Overweight**	6.83	(1.29)	5.20	(1.45)	7.25	(2.17)	5.57	(1.71)	6.70	(4.72)	4.90	(2.85)	5.85	(0.70)
	**Class I obese**	7.41	(2.10)	6.00	(1.61)	7.42	(3.63)	6.52	(2.02)	7.60	(4.06)	5.86	(3.30)	6.38	(0.98)
	**Class II obese**	8.59	(2.97)	5.48	(2.91)	9.69	(4.10)	8.11	(3.20)	9.45	(7.24)	5.91	(4.50)	7.44	(1.49)
	**Class III obese**	8.45	(3.94)	8.71	(3.22)	11.65	(5.34)	9.29	(2.99)	8.16	(11.40)	6.05	(9.96)	9.10	(1.75)
	**Overall**	6.94	(0.83)	5.67	(0.79)	7.67	(1.60)	6.81	(0.98)	7.07	(2.20)	5.15	(1.56)	6.24	(0.44)
**30–39**	**Underweight**	5.45	(4.29)	4.49	(3.80)	–	(–)	7.07	(7.22)	–	(–)	–	(–)	5.83	(2.60)
	**Normal-weight**	4.85	(0.93)	3.59	(0.67)	5.18	(2.04)	3.96	(1.29)	4.49	(2.60)	3.55	(1.95)	4.12	(0.49)
	**Overweight**	4.26	(0.61)	3.19	(0.78)	5.42	(1.51)	3.78	(1.15)	4.39	(2.04)	3.57	(1.81)	3.85	(0.41)
	**Class I obese**	4.60	(0.85)	3.55	(0.91)	5.32	(1.65)	4.45	(1.32)	4.75	(3.16)	3.49	(2.45)	4.17	(0.50)
	**Class II obese**	5.66	(1.49)	4.16	(1.31)	6.03	(2.74)	4.80	(1.59)	4.87	(3.69)	3.87	(2.93)	4.83	(0.74)
	**Class III obese**	6.17	(2.33)	5.63	(1.56)	6.29	(3.86)	5.67	(1.75)	5.63	(4.63)	3.92	(5.37)	5.97	(1.01)
	**Overall**	4.83	(0.42)	3.84	(0.41)	5.73	(0.88)	4.67	(0.59)	4.88	(1.26)	3.52	(1.00)	4.48	(0.24)
**40–49**	**Underweight**	5.75	(5.20)	5.87	(6.28)	–	(–)	3.09	(8.75)	–	(–)	4.72	(9.21)	5.90	(3.29)
	**Normal-weight**	4.12	(0.88)	3.43	(0.51)	4.74	(1.99)	3.82	(1.08)	4.51	(2.26)	2.89	(1.68)	3.65	(0.40)
	**Overweight**	3.97	(0.53)	3.27	(0.61)	4.62	(1.24)	3.55	(0.94)	3.96	(1.71)	3.09	(1.62)	3.70	(0.34)
	**Class I obese**	4.18	(0.78)	3.43	(0.67)	4.90	(1.63)	4.08	(1.08)	4.22	(2.63)	3.43	(1.95)	3.83	(0.43)
	**Class II obese**	5.29	(1.21)	4.10	(1.04)	5.17	(3.45)	4.68	(1.94)	3.66	(4.11)	4.07	(2.66)	4.62	(0.67)
	**Class III obese**	5.23	(1.90)	4.70	(1.22)	5.43	(4.89)	5.04	(1.99)	6.34	(5.51)	4.82	(3.27)	5.00	(0.85)
	**Overall**	4.52	(0.36)	3.82	(0.29)	5.00	(0.85)	4.44	(0.54)	4.49	(1.09)	3.68	(0.87)	4.23	(0.19)
**50–59**	**Underweight**	3.84	(6.32)	2.82	(3.11)	4.39	(10.37)	5.27	(9.80)	–	(–)	–	(–)	4.61	(2.55)
	**Normal-weight**	2.81	(0.86)	2.30	(0.46)	3.92	(1.72)	2.89	(1.15)	3.21	(2.18)	1.96	(1.43)	2.54	(0.35)
	**Overweight**	2.80	(0.55)	2.10	(0.48)	3.34	(1.33)	2.57	(0.98)	2.89	(1.66)	2.12	(1.45)	2.44	(0.32)
	**Class I obese**	2.80	(0.79)	2.41	(0.64)	3.10	(1.97)	2.78	(1.16)	2.83	(3.07)	2.24	(2.19)	2.70	(0.43)
	**Class II obese**	3.55	(1.69)	2.66	(1.10)	3.22	(4.44)	2.94	(2.04)	1.70	(8.51)	2.43	(4.33)	2.97	(0.77)
	**Class III obese**	3.36	(2.66)	3.59	(1.60)	2.66	(8.02)	3.60	(2.51)	2.91	(11.35)	3.88	(3.57)	3.49	(1.12)
	**Overall**	3.11	(0.36)	2.54	(0.27)	3.59	(0.91)	3.19	(0.60)	2.87	(1.14)	2.40	(0.87)	2.86	(0.18)
**60+**	**Underweight**	0.84	(2.27)	0.32	(0.62)	–	(–)	1.07	(2.59)	7.43	(8.42)	0.30	(2.60)	0.33	(0.56)
	**Normal-weight**	0.88	(0.37)	0.27	(0.18)	1.48	(0.99)	0.99	(0.60)	1.15	(1.28)	1.15	(0.79)	0.47	(0.16)
	**Overweight**	1.05	(0.35)	0.51	(0.23)	1.03	(0.93)	0.80	(0.65)	1.40	(1.51)	0.75	(1.18)	0.67	(0.19)
	**Class I obese**	1.33	(0.74)	0.85	(0.39)	1.79	(1.83)	1.38	(0.92)	2.09	(3.27)	1.88	(2.19)	1.05	(0.31)
	**Class II obese**	2.92	(1.69)	0.90	(0.88)	0.50	(5.22)	2.32	(1.71)	1.74	(4.48)	1.82	(7.38)	1.44	(0.72)
	**Class III obese**	2.08	(2.99)	1.58	(1.31)	0.20	(5.35)	2.89	(3.26)	–	(–)	–	(–)	1.86	(1.04)
	**Overall**	1.31	(0.23)	0.62	(0.14)	1.38	(0.62)	1.45	(0.38)	1.29	(0.88)	1.33	(0.61)	0.89	(0.10)
**Overall**		2.67	(0.16)	0.79	(0.11)	3.45	(0.35)	2.25	(0.22)	3.39	(0.55)	2.11	(0.35)	1.58	(0.08)

Notes: a. Bootstrapping means and standard errors in parentheses are presented; –: no observations in the sample.

ORDs appeared to decrease life years with increasing degree of obesity. Overall, the class III obese lost 4.32 life years (from 0.20 to 11.65 life years across race-gender-age groups as shown in Table 2); the class II obese lost 3.17 life years (from 0.5 to 9.69 life years); the class I obese lost about 2.17 life years (from 0.85 to 7.42 years); and the overweight lost 1.31 life years (from 0.51 to 7.25 life years, depending on their age, gender, and race).

In terms of gender and race, black males lost the most life years to ORDs (3.45 years) across all ages and all degrees of obesity, followed by males other than blacks and whites (3.39 years), white males (2.67 years), black females (2.25 years), females other than blacks and whites (2.11 years), and, lastly, white females (0.79 years). ORDs appeared to lessen the most life years of class III obese black males aged 40 years and under (11.65 years for ages 29 and below and 6.29 years for ages 30 to 39).

The pattern of the predicted life years lost associated with ORDs in the sensitivity analysis was only marginally different from the main analysis (Table 2), though most of the standard errors in sensitivity analysis were larger. We present the sensitivity results in Table S3 in Appendix S1.

### Hazard Ratios for Death

**Figure 2:**
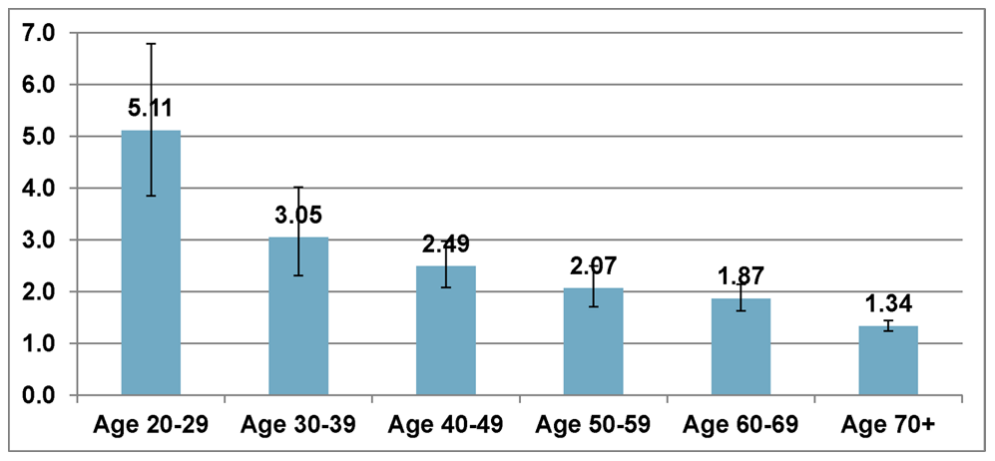
Marginal effect of obesity-related diseases on mortality rate for each age group.

Figure 2 shows the marginal effects of ORDs on hazard rate. An additional ORD was associated with an increase in risk of death for every age group, though mortality risk was more severe for younger individuals: 5.11 [95% CI: 3.85–6.79] for ages 20 to 29; 3.05 [95% CI: 2.31–4.02] for ages 30 to 39; 2.49 [95% CI: 2.09–2.98] for ages 40 to 49; 2.07 [95% CI: 1.71–2.50] for ages 50 to 59; 1.87 [95% CI: 1.63–2.14] for ages 60 to 69; and 1.34 [95% CI: 1.24–1.44] for ages 70 and above.

**Figure 3:**
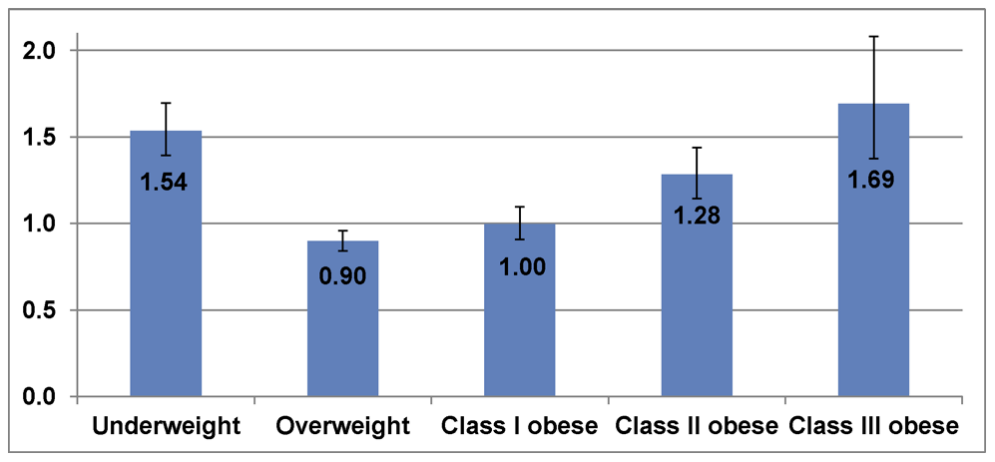
Hazard ratios for death for each BMI classification (reference: the normal-weight).

To compare with those in previous studies, the hazard ratios for death for each BMI classification were computed (see the parameter estimates for each BMI classification in the final model in Table S2 in Appendix S1). We found that the underweight, class II, and class III obese had higher mortality rates than the normal-weight (Figure 3). The class III obese had the highest hazard ratio (1.69 [95% CI: 1.37–2.08]) compared to normal-weight people, followed by the underweight (1.54 [95% CI: 1.39–1.70]), and the class II obese (1.28 [95% CI: 1.14–1.44]). The overweight (0.90 [95% CI: 0.84–0.96]) and class I obese (0.997 [95% CI: 0.91–1.09]) had lower death rates than the normal-weight, but the latter was not statistically significant.

## Discussion

We investigated the relationship between ORDs and mortality by using the MPH model controlling for both observed and unobserved individual heterogeneity. Using data from NHIS 1997 to 2000 and NHIS Linked Mortality Files to estimate the parameters in the MPH model, we predicted life years lost associated with ORDs based on the estimates in the model, using simulated race-gender-age-BMI classification-ORD status cohorts of U.S. non-smoking adults.

We confirmed that non-smoking adults with ORDs had higher mortality. Depending on the age group, an additional ORD increased risk of death by a range of 34% to 411%. This finding is consistent with Kuk et al. [28], who used the Edmonton Obesity Staging System (EOSS) to measure ORDs and found that the hazard rate for people at stage 2 of EOSS was 119% higher than the hazard rate for people at stage 1, and the hazard rate for people at stage 3 of EOSS was 35% higher than the hazard rate for people at stage 2. However, a direct comparison is difficult because the data, models, and measurements of ORDs are different.

Comparing and contrasting our study with previous studies that explored the relationship between BMI/obesity and mortality and took ORDs into account, we found that adults who belonged to overweight and class I obese classifications had lower mortality rates, while adults who belonged to underweight, class II and III obese classifications had higher mortality rates than normal-weight people, other things being equal. Consistent with previous findings in the literature [20], [21], [23], [26], [56], our findings showed that higher degrees of obesity and underweight were associated with higher mortality. But deviating from Berrington de Gonzalez et al. [20] and others [19],[21], our BMI-mortality association approximated a U-shaped relationship with the lowest mortality rate in the overweight classification [23], [24], [26], [56], while theirs approximated a J-shaped relationship with the lowest mortality rate in the normal-weight classification. This deviation can be explained by at least three reasons: (i) our study adjusted for ORDs, and hence, the nadir for death rates shifted slightly to the right in the range of overweight; (ii) only whites were included in the sample of Berrington de Gonzalez et al. [20], eliminating ethnic variation, which is known to be a key determinant of obesity-mortality relationships; (iii) unobserved heterogeneity was not controlled for in their analyses; thus, the sample was selected toward survivors.

The predicted life years lost associated with ORDs decreased with age. ORDs were expected to shorten the lifespan of people in their 20 s by more than 5 years, while people in their 60 s were predicted to lose just under 1 year of life. Fontaine et al. [14] also found a similar trend in the context of life years lost to obesity. Using U.S. non-smoking adults aged 40 to 49 years as an example to illustrate percentage of the life years lost associated with ORDs, we found that on average a 45-year-old U.S. non-smoking class III obese black male with ORDs (compared with his counterpart without ORDs) was expected to lose 5.43 years of life, which translates to a 7.5% reduction in the total life years (72.78 years). Of the same age and same degree of obesity, white males lost 5.23 life years (a 6.8% reduction in total life years), followed by black females (5.04 years, a 6.5% reduction in life years), and white females (4.70 years, a 5.8% reduction in life years). The total life years were obtained by averaging 4-year life expectancy for the target populations from the U.S. Life Tables [57]–[60]. Based on these average life years lost estimates, total life years lost associated with ORDs for U.S. non-smoking adults aged 40 to 49 years was estimated to be 914,573 for white males (n = 174,821); 1,324,105 for white females (n = 281,679); 189,847 for black males (n = 34,967); and 501,745 for black females (n = 99,556).

The predicted life years lost associated with ORDs also increased with degree of obesity. Although our final model did not directly support the finding that the death rates associated with ORDs were increasing with the degree of obesity, this increase was possibly driven by the fact that ORDs were more prevalent in people with higher degrees of obesity, and an increased risk of mortality was associated with an additional ORD (Figure 2).

Our analysis has several strengths. First, our analysis focused on ORDs and examined their association with mortality and life years, an association that has not been well studied. Second, unlike most studies, our study controlled for unobserved heterogeneity, which captures the effects of unavailable variables in the data; as a result, our estimations are more precise, and our prediction of life years lost associated with ORDs is more reliable. Third, we incorporated the degree of obesity confounding the analysis when exploring the relationship between ORDs and mortality. Overall, this study not only advances in methodology, but provides a different perspective on the relationship between ORDs and mortality, which will inform the future analyses of any weight-loss intervention, ranging from physical activity programs to bariatric surgery, including, but not limited to, cost-effectiveness analyses.

Yet, our study has several limitations. First, using ORD counts prevented us from differentiating the effects of different diseases. Second, due to data constraints, the ORDs this paper targeted were a subset of obesity-related comorbidities. Third, the data that we used are cross-sectional, and our model is not time-varying and does not capture the dynamics of disease evolution, which limits its projection capability on life years lost [46]. For example, smoking status might change over time for the younger cohorts in particular, which could add variation to the relationships. Fourth, some studies [61]–[63] suggest that different weight predictors of mortality, e.g., waist-hip ratio, waist circumference, or fitness, might be better measures than BMI. Even if they are not, incorporating them would have made the analysis more comprehensive. However, these data were not available in the NHIS datasets.

### Conclusion

Our results confirm that being obese or underweight increased risk of mortality. Furthermore, our study suggests that the ORDs included in our study – coronary heart disease, hypertension, diabetes, and stroke – increased chances of dying and decreased life years by 0.2 to 11.7 years depending on gender, race, BMI classification, and age. The life years lost associated with ORDs was more pronounced for younger, black, male, and more obese adults than for older, white, female, and less obese adults.

This conclusion not only conveys a message that these populations are more vulnerable to ORDs, but it also informs policy makers that public health initiatives should put more emphasis on the prevention of obesity and obesity-related comorbidities for these populations. More importantly, future studies should investigate how different ORDs separately observed at different life stages impact life years lost.

## Supporting Information

Appendix S1 Table S1–S3: Considered model specifications and parametric assumptions; Estimation results: final model; Predicted life years lost associated with obesity-related diseases for U.S. non-smoking adults, 1997–2000: sensitivity analysis.
(DOCX)
