# Supplementary material for: Life Years Lost Associated with Obesity-Related Diseases for U.S. Non-Smoking Adults
Source: PLoS One. 2013 Jun 18;8(6):e66550. doi: 10.1371/journal.pone.0066550 (PMC3688902; doi:10.1371/journal.pone.0066550)
Supplement: Appendix S1 — Table S1–S3: Considered model specifications and parametric assumptions; Estimation results: final model; Predicted life years lost associated with obesity-related diseases for U.S. non-smoking adults, 1997–2000: sensitivity analysis. (DOCX) [file pone.0066550.s001.docx]

**APPENDIX S1 for**

**Life years lost associated with obesity-related diseases for U.S. non-smoking adults**

1. **Overview**

Our study used the mixed proportional hazard (MPH) model [[1](#_ENREF_1)], which expands on the Cox proportional hazards (PH) model [[2](#_ENREF_2)]. The MPH model is a proportional hazards model with multiplicative unobserved heterogeneity. The importance of accounting for unobserved heterogeneity in the frailty model has been previously studied [[1](#_ENREF_1),[3-5](#_ENREF_3)]. It has been noted that individuals with relatively high hazard rates for unobserved reasons die earlier so that samples of survivors are selected. Therefore, the estimated hazard rate of the selected sample increases slower with duration than that of a random sample. In addition, the estimated partial effects of covariates on the hazard rate of the selected sample are smaller in terms of scale than those on the hazard rate of a random sample [[6](#_ENREF_6)]. Consequently, the estimated hazard ratios tend to be biased, and the direction of the bias depends on the scale of the down-sized estimates.

1. **MPH Model Specifications and Estimation**

Let uppercase letters denote random variables and lowercase letters denote the realizations of the corresponding random variables. Denote $\theta$ as the hazard rate, i.e., transition rate to death. For each individual, the hazard rate was determined by the following equation:

$\theta(t|v,\mathbf{x}',\mathbf{\beta}')=\lambda(t)\cdot v\cdot\exp(\mathbf{x}'\mathbf{\beta})$,

where $\lambda(t)$ is the baseline hazard; $V$ is the unobserved heterogeneity, which has the distribution $G$; $\beta$ is a vector of parameters to be estimated; and $x$ is a vector of covariates, which consist of an intercept, BMI information (BMI classifications, inverted BMI, BMI level, or quadratic form of BMI), high school graduates, and binary variables for males, whites, blacks, alcohol consumption (whether an individual had had no more than 12 drinks of any type of alcoholic beverage in the individual’s entire life upon survey), physical activity (whether an individual was engaged in modest or vigorous physical activities for 10 minutes at least once per week). Information on obesity-related diseases was either dichotomized by disease status (whether an individual has at least one obesity-related disease) or counted by the number of obesity-related diseases. The outcome variable is age at death or age at censor, denoted by $T$. Further assume that the baseline hazard, $\lambda(t)$, is piecewise constant [[7-9](#_ENREF_7)]:

$$\lambda(t)=\left\{\begin{matrix} \lambda_{1} & & t\leq30 \\ \lambda_{2} & & t\in(30,35] \\ \lambda_{3} & & t\in(35,40] \\ \vdots& & \vdots\\ \lambda_{13} & & t\in(85,90] \\ \lambda_{14} & & t>90, \end{matrix}\right.$$

where we used 14 parameters, each representing the time-dependent risk for the individual in one particular interval of age. We normalized $\lambda_{1}$ ($\lambda_{1}=1$). The unobserved heterogeneity, $V$, was assumed to have a gamma distribution based on Abbring and van den Berg’s findings [[3](#_ENREF_3)],[[1]](#footnote-1) and we restrict that $E(V)=1$ for identification purpose [[14](#_ENREF_14)]. Therefore, the gamma distributed $V$ has one parameter to be estimated, denoted $k$. The probability density function of $V$is as follows:

$$g(v)=\frac{k^{k}}{\Gamma(k)}v^{k-1}e^{-kv},$$

where $k>0$ and $\Gamma\left( k \right)=\left( k-1 \right)!$. Alternatively, we considered a bivariate distributed $V$, where $V$takes on two values – $v^{a}$ and $v^{b}$ – with probability $p$ and $1-p$, respectively [[5](#_ENREF_5)].

We used quasi-maximum likelihood estimation, which adjusts for the complex sampling design in the NHIS datasets [[15](#_ENREF_15)] to estimate the parameters and their standard errors in the MPH model.

1. **Estimation Results**

We tested different model specifications and parametric assumptions. For the parametric specification, we also assumed a bivariate distributed unobserved heterogeneity and changed the number of parameters for the baseline hazard. Different combinations of model specifications and parametric assumptions were performed. All model specifications considered in our study are listed in Table S1. Different functional forms for BMI were tested: linear for specification I, quadratic [[16](#_ENREF_16),[17](#_ENREF_17)] for specification II and IV, and inverted BMI [[17-19](#_ENREF_17)] for specification III. We rescaled covariates as needed to ensure feasible initial solutions in the optimization routine, and the same rescaling was made throughout the sensitivity analyses for direct comparisons.

Due to the parametric specification of unobserved heterogeneity in our study, we also tested different parametric specifications of the unobserved heterogeneity, $V$. Assume that $V$ has a bivariate distribution (specifications VI and VII), taking on two values – $v^{a}$ and $v^{b}$ – with probability $p$ and $1-p$, respectively [[5](#_ENREF_5)]. The parameter estimates had the same signs as those in the baseline case. But the estimates for $p$ in all specifications appeared to converge to the bound. We also increased the number of mass points for $V$, and the results appeared to be similar. In specification V, we decreased the number of baseline hazards. The results showed a more consistent pattern of increasing likelihood of death as age increases. We also generated different combinations of interaction terms associated with the number of ORDs (specification IX, XI – XIII) or ORD status (specification X).

We used a weighted Akaike information criterion (WAIC) [[20](#_ENREF_20)] to determine the final model, and the parameter estimates in the final model were used in life years lost simulations. A full set of parameter estimates is presented in Table S2. The estimates suggested that keeping other factors fixed, males, blacks, less educated, or physically less active individuals have higher mortality risks. Surprisingly, people who had less than 12 drinks in their entire life posed risk to mortality, *ceteris paribus*. The marginal effect of ORDs depends on the individual’s age upon survey. The estimates show that the ORDs are positively associated with an individual’s mortality no matter which age group the individual belongs in.

In terms of the degree of obesity, we found that individuals who belonged to underweight, class II, and class III obese classifications had higher hazard rates than people who belonged to normal-weight classification. The overweight (and class I obese) had lower hazard rates than the normal-weight. The seemingly counterintuitive result can be rationalized by the fact that we adjusted for the number of ORDs along with other confounders. Therefore, keeping the number of ORDs fixed, the overweight and the mildly obese were not at as high of a risk as they were when the number of ORDs was not controlled for. However, the class II and III obese were still found to be at a higher risk of mortality even though the number of ORDs was adjusted.

The estimates of baseline hazards showed positive duration dependence in both models, i.e., $\hat{\lambda}_{2}<\hat{\lambda}_{3}<\cdots<\hat{\lambda}_{13}$, except for $\hat{\lambda}_{14}$,[[2]](#footnote-2) indicating that the likelihood of death increased with age. The estimate for the parameter of the gamma distributed unobserved heterogeneity was 37.55.

1. **Prediction of Life Years**

We divided our sample into subgroups based on different combinations of race, gender, age, and BMI classification. For each subgroup, we selected a sample with replacement, according to the sampling weights [[21](#_ENREF_21)]. Using the parameter estimates in the final model, we obtained the estimated survival densities:

where is the estimated cumulative baseline hazard. We then used the inverse transform technique to simulate random life years, which follow the cumulative density distributions. In order to compute the means and standard errors, we resampled the subpopulation 1,000 times, following the aforementioned steps.

1. **Sensitivity Analyses**

To explore parameter uncertainty in the simulation, we performed a probabilistic sensitivity analysis. We first sampled the parameters from the distribution of our quasi-maximum likelihood estimators [[15](#_ENREF_15)]. For each set of parameters, we simulated life years for each subgroup and computed the life years lost associated with ORDs for each subgroup. We repeated this process 1,000 times and computed the means and standard errors. We present the sensitivity analysis results in Table S3.

The pattern of the predicted life years lost associated with ORDs in the sensitivity analysis is only marginally different from the main analysis (Table 2), though most of the standard errors in sensitivity analysis are larger. Consistent with the baseline life years lost prediction, ORDs appeared to decrease life years with increasing degree of obesity. In addition, the younger an adult developed ORDs, the more life years were lost associated with the comorbid conditions. In terms of gender and race, black males appeared to lose the most life years across all ages, followed by white males, black females, and white females.

1. **References**

1. Lancaster T (1979) Econometric Methods for the Duration of Unemployment. Econometrica 47: 939-956.

2. Cox DR (1972) Regression Models and Life-Tables. Journal of the Royal Statistical Society Series B-Statistical Methodology 34: 187-&.

3. Abbring JH, Van den Berg GJ (2007) The unobserved heterogeneity distribution in duration analysis. Biometrika 94: 87-99.

4. Vaupel JW, Manton KG, Stallard E (1979) Impact of Heterogeneity in Individual Frailty on the Dynamics of Mortality. Demography 16: 439-454.

5. Heckman JJ, Singer B (1984) Econometric Duration Analysis. Journal of Econometrics 24: 63-132.

6. Gerard J VdB (2001) Chapter 55 Duration models: specification, identification and multiple durations. In: Heckman JJ, Leamer E, editors. Handbook of Econometrics: Elsevier. pp. 3381-3460.

7. Gray RJ (1994) A Bayesian analysis of institutional effects in a multicenter cancer clinical trial. Biometrics 50: 244-253.

8. Yamaguchi T, Ohashi Y, Matsuyama Y (2002) Proportional hazards models with random effects to examine centre effects in multicentre cancer clinical trials. Stat Methods Med Res 11: 221-236.

9. Han A, Hausman JA (1990) Flexible Parametric-Estimation of Duration and Competing Risk Models. Journal of Applied Econometrics 5: 1-28.

10. Nielsen GG, Gill RD, Andersen PK, Sorensen TIA (1992) A Counting Process Approach to Maximum-Likelihood-Estimation in Frailty Models. Scandinavian Journal of Statistics 19: 25-43.

11. Murphy SA (1994) Consistency in a Proportional Hazards Model Incorporating a Random Effect. Annals of Statistics 22: 712-731.

12. Murphy SA (1995) Asymptotic Theory for the Frailty Model. Annals of Statistics 23: 182-198.

13. Rahgozar M, Faghihzadeh S, Rouchi GB, Peng Y (2008) The power of testing a semi-parametric shared gamma frailty parameter in failure time data. Stat Med 27: 4328-4339.

14. Elbers C, Ridder G (1982) True and Spurious Duration Dependence - the Identifiability of the Proportional Hazard Model. Review of Economic Studies 49: 403-409.

15. Sakata S (2002) Quasi-maximum likelihood estimation with complex survey data. Mimeo.

16. Heo M, Faith MS, Mott JW, Gorman BS, Redden DT, et al. (2003) Hierarchical linear models for the development of growth curves: an example with body mass index in overweight/obese adults. Stat Med 22: 1911-1942.

17. Fontaine KR, Redden DT, Wang C, Westfall AO, Allison DB (2003) Years of life lost due to obesity. JAMA 289: 187-193.

18. Allison DB, Faith MS, Heo M, Kotler DP (1997) Hypothesis concerning the U-shaped relation between body mass index and mortality. Am J Epidemiol 146: 339-349.

19. Durazo-arvizu R, McGee D, Li Z, Cooper R (1997) Establishing the nadir of the body mass index-mortality relationship: a case study. J Am Stat Assoc 92: 1,312-319.

20. Hens N, Aerts M, Molenberghs G (2006) Model selection for incomplete and design-based samples. Stat Med 25: 2502-2520.

21. Centers for Disease Control and Prevention (2000) Design and estimation for the National Health Interview Survey, 1995-2004. Vital Health Stat 2: 1-31.

Table S1. Considered model specifications and parametric assumptions

| **Model** | **Covariates** | $V$ | **Baseline hazard** |
| --- | --- | --- | --- |
| **I** | Male^a^, white^a^, black^a^, high school^a^, BMI, ORDs, no alcohol^a^, physical activity^a^, age^b^, age squared^c^ | Gamma | Piecewise constant (14 parameters) |
| **II** | Male^a^, white^a^, black^a^, high school^a^, BMI^b^, BMI squared^c^, ORDs, no alcohol^a^, physical activity^a^, age^b^, age squared^c^ | Gamma | Piecewise constant (14 parameters) |
| **III** | Male^a^, white^a^, black^a^, high school^a^, inverted BMI, ORDs, no alcohol^a^, physical activity^a^, age^b^, age squared^c^ | Gamma | Piecewise constant (14 parameters) |
| **IV** | Male^a^, white^a^, black^a^, high school^a^, BMI, BMI squared^c^, ORDs, no alcohol^a^, physical activity^a^, age^b^, age^b^×ORDs | Gamma | Piecewise constant (14 parameters) |
| **V** | Male^a^, white^a^, black^a^, high school^a^, BMI^b^, BMI squared, ORDs, no alcohol^a^, physical activity^a^, age^b^, age squared^c^ | Gamma | Piecewise constant (12 parameters) |
| **VI** | Male^a^, white^a^, black^a^, high school^a^, inverted BMI, ORDs, no alcohol^a^, physical activity^a^, age^b^, age squared^c^ | Bivariate | Piecewise constant (14 parameters) |
| **VII** | Male^a^, white^a^, black^a^, high school^a^, underweight^a^, overweight^a^, class I obese^a^, class II obese^a^, class III obese^a^, ORD^a^, no alcohol^a^, physical activity^a^ | Bivariate | Piecewise constant (14 parameters) |
| **VIII** | Male^a^, white^a^, black^a^, high school^a^, BMI^b^, ORDs, no alcohol^a^, physical activity^a^, age2029^a^, age3039^a^, age4049^a^, age5059^a^, age6069^a^, age70+^a^, age2029×ORDs, age3039×ORDs, age4049×ORDs, age5059×ORDs, age6069×ORDs, age70+×ORDs, white×ORDs, black×ORDs, male×ORDs | Gamma | Piecewise constant (14 parameters) |
| **IX** | Male^a^, white^a^, black^a^, high school^a^, underweight^a^, overweight^a^, class I obese^a^, class II obese^a^, class III obese^a^, ORD^a^, no alcohol^a^, physical activity^a^, age2029^a^, age3039^a^, age4049^a^, age5059^a^, age6069^a^, age70+^a^, age2029×ORDs, age3039×ORDs, age4049×ORDs, age5059×ORDs, age6069×ORDs, age70+×ORDs, underweight×ORDs, overweight×ORDs, class I obese×ORDs, class II obese×ORDs, class III obese×ORDs | Gamma | Piecewise constant (14 parameters) |
| **X** | Male^a^, white^a^, black^a^, high school^a^, BMI, ORD^a^, no alcohol^a^, physical activity^a^, age2029^a^, age3039^a^, age4049^a^, age5059^a^, age6069^a^, age70+^a^, age2029×ORD, age3039×ORD, age4049×ORD, age5059×ORD, age6069×ORD, age70+×ORD | Gamma | Piecewise constant (14 parameters) |
| **XI** | Male^a^, white^a^, black^a^, high school^a^, underweight^a^, overweight^a^, class I obese^a^, class II obese^a^, class III obese^a^, ORDs, no alcohol^a^, physical activity^a^, age2029^a^, age3039^a^, age4049^a^, age5059^a^, age6069^a^, age70+^a^, age2029×ORDs, age3039×ORDs, age4049×ORDs, age5059×ORDs, age6069×ORDs, age70+×ORDs, underweight×ORDs, overweight×ORDs, class I obese×ORDs, class II obese×ORDs, class III obese×ORDs, white×ORDs, black×ORDs, male×ORDs | Gamma | Piecewise constant (14 parameters) |
| **XII** | Male^a^, white^a^, black^a^, high school^a^, underweight^a^, overweight^a^, class I obese^a^, class II obese^a^, class III obese^a^, ORDs, no alcohol^a^, physical activity^a^, age2029^a^, age3039^a^, age4049^a^, age5059^a^, age6069^a^, age70+^a^, underweight×ORDs, overweight×ORDs, class I obese×ORDs, class II obese×ORDs, class III obese×ORDs | Gamma | Piecewise constant (14 parameters) |
| **XIII** | Male^a^, white^a^, black^a^, high school^a^, underweight^a^, overweight^a^, class I obese^a^, class II obese^a^, class III obese^a^, ORDs, no alcohol^a^, physical activity^a^, age2029^a^, age3039^a^, age4049^a^, age5059^a^, age6069^a^, age70+^a^, white×ORDs, black×ORDs, male×ORDs | Gamma | Piecewise constant (14 parameters) |

a. Binary variable: 1 indicates the individual belongs to that category; and 0 otherwise. b. Realizations were divided by 10. c. Realizations were divided by 100.

Table S2. Estimation results:^a^ final model

| **Outcome: mortality** | |  |  |  |  | |  |
| --- | --- | --- | --- | --- | --- | --- | --- |
| **Intercept** | -40.381 | | (8.703) | **Age3039×ORDs** | 1.352 | (0.294) | |
| **Male**^b^ | 0.457 | | (0.035) | **Age4049×ORDs** | 1.150 | (0.290) | |
| **White**^b^ | 0.020 | | (0.059) | **Age5059×ORDs** | 0.964 | (0.362) | |
| **Black**^b^ | 0.248 | | (0.064) | **Age6069×ORDs** | 0.862 | (0.323) | |
| **High school**^b^ | -0.160 | | (0.031) | **Age70+×ORDs** | 0.528 | (0.296) | |
| **Underweight**^b^ | 0.429 | | (0.050) | $\boldsymbol{k}$ | 37.551 | (9.622) | |
| **Overweight**^b^ | -0.108 | | (0.033) | $\boldsymbol{\lambda}_{\boldsymbol{1}}$ | 1.000 | (--) | |
| **Class I obese**^b^ | -0.003 | | (0.048) | $\boldsymbol{\lambda}_{\boldsymbol{2}}$ | 0.524 | (0.074) | |
| **Class II obese**^b^ | 0.249 | | (0.058) | $\boldsymbol{\lambda}_{\boldsymbol{3}}$ | 0.795 | (0.178) | |
| **Class III obese**^b^ | 0.526 | | (0.105) | $\boldsymbol{\lambda}_{\boldsymbol{4}}$ | 1.508 | (0.212) | |
| **ORDs**^c^ | -0.237 | | (0.303) | $\boldsymbol{\lambda}_{\boldsymbol{5}}$ | 2.524 | (0.431) | |
| **No alcohol**^b^ | 0.169 | | (0.033) | $\boldsymbol{\lambda}_{\boldsymbol{6}}$ | 3.138 | (0.553) | |
| **Physical activity**^b^ | -0.333 | | (0.030) | $\boldsymbol{\lambda}_{\boldsymbol{7}}$ | 4.129 | (0.787) | |
| **Age2029**^b^ | 31.508 | | (8.763) | $\boldsymbol{\lambda}_{\boldsymbol{8}}$ | 8.273 | (1.657) | |
| **Age3039**^b^ | 31.622 | | (8.742) | $\boldsymbol{\lambda}_{\boldsymbol{9}}$ | 13.030 | (3.494) | |
| **Age4049**^b^ | 31.958 | | (8.651) | $\boldsymbol{\lambda}_{\boldsymbol{10}}$ | 26.334 | (6.027) | |
| **Age5059**^b^ | 31.851 | | (8.534) | $\boldsymbol{\lambda}_{\boldsymbol{11}}$ | 65.854 | (16.251) | |
| **Age6069**^b^ | 32.175 | | (8.511) | $\boldsymbol{\lambda}_{\boldsymbol{12}}$ | 151.046 | (35.953) | |
| **Age70+**^b^ | 31.622 | | (8.459) | $\boldsymbol{\lambda}_{\boldsymbol{13}}$ | 502.771 | (129.300) | |
| **Age2029×ORDs** | 1.869 | | (0.402) | $\boldsymbol{\lambda}_{\boldsymbol{14}}$ | 330.210 | (84.246) | |
| **WAIC** | 34,062 | | | | | | |

1. Standard errors are in parentheses.
2. Binary variable: 1 indicates the individual belongs to that category; and 0 otherwise.
3. ORDs: the number of obesity-related diseases.

Table S3. Predicted life years lost associated with obesity-related diseases for U.S. non-smoking adults, 1997-2000: sensitivity analysis^a^

|  |  | **White male** | | **White female** | | **Black male** | | **Black female** | | **Other male** | | **Other female** | |
| --- | --- | --- | --- | --- | --- | --- | --- | --- | --- | --- | --- | --- | --- |
| **29-** | **Underweight** | 7.21 | (11.04) | 7.00 | (8.54) | -- | (--) | 6.60 | (10.02) | 9.22 | (13.40) | 6.02 | (9.83) |
|  | **Normal-weight** | 6.19 | (6.67) | 5.73 | (5.35) | 8.36 | (6.27) | 7.62 | (4.10) | 6.22 | (8.05) | 4.94 | (7.33) |
|  | **Overweight** | 6.86 | (4.84) | 5.37 | (5.19) | 7.41 | (4.83) | 5.86 | (6.13) | 7.11 | (6.52) | 5.44 | (6.01) |
|  | **Class I obese** | 7.29 | (5.03) | 6.28 | (4.81) | 7.53 | (5.51) | 7.06 | (5.12) | 7.68 | (7.49) | 6.67 | (5.86) |
|  | **Class II obese** | 8.39 | (5.45) | 5.58 | (6.30) | 8.68 | (7.65) | 7.74 | (5.64) | 8.09 | (12.05) | 5.71 | (7.67) |
|  | **Class III obese** | 7.90 | (5.53) | 8.43 | (5.21) | 10.45 | (6.90) | 8.52 | (6.79) | 6.70 | (13.65) | 7.12 | (12.16) |
| **30-39** | **Underweight** | 5.01 | (5.23) | 4.74 | (5.13) | -- | (--) | 6.55 | (8.14) | -- | (--) | -- | (--) |
|  | **Normal-weight** | 4.82 | (3.35) | 4.25 | (3.21) | 4.98 | (3.64) | 4.32 | (3.27) | 4.43 | (3.91) | 4.09 | (3.68) |
|  | **Overweight** | 4.46 | (3.10) | 3.86 | (3.10) | 5.25 | (3.61) | 4.29 | (3.22) | 4.55 | (3.76) | 4.13 | (3.57) |
|  | **Class I obese** | 4.68 | (3.10) | 3.97 | (3.11) | 5.12 | (3.49) | 4.68 | (3.45) | 4.56 | (4.28) | 4.03 | (3.76) |
|  | **Class II obese** | 5.36 | (3.54) | 4.44 | (3.28) | 5.58 | (4.16) | 4.81 | (3.41) | 4.84 | (4.68) | 4.02 | (4.13) |
|  | **Class III obese** | 5.55 | (3.66) | 5.39 | (3.70) | 5.52 | (4.71) | 5.42 | (3.59) | 5.28 | (5.69) | 4.13 | (6.20) |
| **40-49** | **Underweight** | 4.86 | (5.73) | 4.92 | (6.51) | -- | (--) | 2.95 | (9.62) | -- | (--) | 4.14 | (9.18) |
|  | **Normal-weight** | 3.75 | (2.37) | 3.49 | (2.44) | 4.24 | (2.99) | 3.62 | (2.50) | 4.02 | (3.09) | 2.95 | (2.65) |
|  | **Overweight** | 3.68 | (2.30) | 3.38 | (2.42) | 4.07 | (2.57) | 3.47 | (2.41) | 3.75 | (2.87) | 3.30 | (2.74) |
|  | **Class I obese** | 3.77 | (2.31) | 3.45 | (2.40) | 4.21 | (2.75) | 3.83 | (2.53) | 3.64 | (3.47) | 3.51 | (2.97) |
|  | **Class II obese** | 4.45 | (2.68) | 3.83 | (2.55) | 4.17 | (3.77) | 4.17 | (2.93) | 3.06 | (4.52) | 3.89 | (3.52) |
|  | **Class III obese** | 4.35 | (2.76) | 4.15 | (2.54) | 4.46 | (5.03) | 4.39 | (2.94) | 5.74 | (6.59) | 4.52 | (4.16) |
| **50-59** | **Underweight** | 2.91 | (6.13) | 2.74 | (3.48) | 3.67 | (10.57) | 4.43 | (9.91) | -- | (--) | -- | (--) |
|  | **Normal-weight** | 2.50 | (1.71) | 2.41 | (1.71) | 3.16 | (2.29) | 2.75 | (2.04) | 2.84 | (2.63) | 2.06 | (1.92) |
|  | **Overweight** | 2.58 | (1.68) | 2.31 | (1.72) | 2.91 | (2.10) | 2.61 | (1.90) | 2.56 | (2.25) | 2.31 | (2.24) |
|  | **Class I obese** | 2.44 | (1.63) | 2.51 | (1.81) | 2.63 | (2.37) | 2.66 | (1.98) | 2.63 | (3.28) | 2.19 | (2.53) |
|  | **Class II obese** | 3.04 | (2.21) | 2.62 | (1.90) | 2.53 | (4.38) | 2.56 | (2.49) | 1.97 | (8.50) | 2.35 | (4.56) |
|  | **Class III obese** | 2.56 | (2.99) | 3.14 | (2.30) | 2.60 | (7.79) | 2.93 | (2.78) | 2.60 | (11.02) | 3.09 | (3.77) |
| **60+** | **Underweight** | 0.76 | (2.17) | 0.06 | (0.69) | -- | (--) | 1.78 | (2.88) | 7.58 | (10.15) | 0.98 | (2.66) |
|  | **Normal-weight** | 0.56 | (0.64) | 0.04 | (0.59) | 0.98 | (1.10) | 0.78 | (0.73) | 0.82 | (1.50) | 1.02 | (1.02) |
|  | **Overweight** | 0.73 | (0.65) | 0.33 | (0.60) | 0.43 | (1.32) | 0.50 | (0.96) | 1.09 | (1.59) | 0.61 | (1.43) |
|  | **Class I obese** | 0.88 | (1.00) | 0.67 | (0.63) | 1.14 | (2.01) | 1.10 | (1.06) | 1.76 | (3.39) | 1.55 | (2.39) |
|  | **Class II obese** | 2.42 | (2.38) | 0.58 | (1.31) | 1.81 | (6.00) | 1.93 | (2.27) | 2.64 | (6.75) | 0.60 | (7.31) |
|  | **Class III obese** | 1.48 | (3.00) | 0.96 | (1.48) | 1.03 | (6.74) | 2.54 | (3.84) | -- | (--) | -- | (--) |

a. Means and standard errors in parentheses from probability sensitivity analysis are presented; --: no observations in the sample.

1. In a large class of hazard models with proportional unobserved heterogeneity, the distribution of the heterogeneity among survivors converges to a gamma distribution. And studies that assume gamma heterogeneity include, for example, Nielsen et al.[[10](#_ENREF_10)], Murphy [[11](#_ENREF_11),[12](#_ENREF_12)], Rahgozar et al.[[13](#_ENREF_13)], and so on. [↑](#footnote-ref-1)
2. The lower estimate for $\lambda_{14}$ can be explained by the fact that the number of people who died at an age older than 90 drops to 476 among the 4,017 individuals in the sample who died before 2006 (see Table 1). The positive dependence was more obvious when the number of parameters for the baseline hazard was reduced (Model V in Table S1).


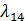


   [↑](#footnote-ref-2)
